# Synergistic muscle coordination of the Paralympic wheelchair tennis champion

**DOI:** 10.3389/fspor.2025.1717457

**Published:** 2026-01-12

**Authors:** Nadaka Hakariya, Takuya Murakami, Keiichi Ishikawa, Daiki Yamasaki, Naotsugu Kaneko, Kimitaka Nakazawa

**Affiliations:** 1 Department of Life Sciences, Graduate School of Arts and Sciences, The University of Tokyo, Tokyo, Japan; 2 Department of Rehabilitation for the Movement Functions, Research Institute of National Rehabilitation Center for Persons with Disabilities, Tokorozawa, Japan; 3 Japan Society for the Promotions Science, Tokyo, Japan

**Keywords:** wheelchair tennis, Paralympic athletes, muscle synergy, impairment-related adaptation, neural plasticity

## Abstract

**Introduction:**

This study aimed to elucidate specific muscle coordination patterns in a Paralympic gold medal wheelchair tennis player. Recent neuroscience has proposed the concept of the “Paralympic brain”, referring to use-dependent and impairment-specific plasticity observed in some Paralympians. Although the present study does not directly assess neural reorganization, this framework provides context for interpreting motor coordination strategies.

**Methods:**

We applied muscle synergy analysis of electromyographic (EMG) activities during the tennis serve to quantify muscle coordination in two male players: one a Paralympic and world champion (P1; osteosarcoma with hip and abdominal resections), and the other nationally ranked sub-elite player (P2; spina bifida with paralysis of the lower limbs and trunk). EMGs from 14 muscles and high-speed video were recorded during three flat serves.

**Results:**

Serve velocity was markedly higher in P1 (167 ± 11.3 km/h) than P2 (80.0 ± 8.9 km/h). Four synergies were identified in P1 and three in P2. A specific synergy (Syn1), dominant in the early takeback phase and involving lower trapezius and triceps brachii, was found only in P1 and may contribute to greater trunk rotation and serve velocity. In addition, the sparseness of muscle synergies was also higher in P1 than in P2.

**Discussion:**

These results indicate that elite serve performance is supported not only by muscular strength but also on specialized neuromuscular coordination strategies optimizing trunk rotation and energy transfer. They may be consistent with broader ideas of use-dependent and impairment-related adaptation described in the “Paralympic brain” framework. This study provides new insights into adapted motor strategies in Paralympic sport, underscoring the role of trunk control and residual function as key factors in training and performance optimization.

## 1 Introduction

Paralympic athletes demonstrate exceptional performance despite physical impairments. Recent neuroscience research has proposed the concept of the “Paralympic brain”, characterized by unique neural reorganization through the interaction between use-dependent and impairment-specific plasticity (1). This synergistic plasticity may contribute to motor control strategies that differ qualitatively from those of able-bodied athletes. While this study does not directly examine neural reorganization, investigating the muscle coordination patterns can deepen our understanding of neuroplasticity and inform rehabilitation and training approaches that aim to optimize residual function (2, 3).

Among Paralympic sports, wheelchair tennis offers a valuable model for studying these mechanisms. The sport requires players to perform powerful and precise upper-limb movements while seated, often without full use of trunk or lower limb function. Particularly, the serve is a ballistic, self-initiated movement conducted from a static posture (closed skill), making it ideal for examining neuromuscular coordination without the confound of wheelchair propulsion. Prior studies have highlighted that residual trunk and lower limb function influence serve speed (4), but the underlying coordination mechanisms remain largely unexplored.

To address this gap, this study conducted a comparative case study involving a wheelchair tennis player who is a Paralympic gold medalist and another athlete with a more severe disability, yielding insights into use-dependent and impairment-related muscle coordination strategies in wheelchair tennis. Measuring such an athlete is particularly meaningful from a scientific standpoint, as gold medalists are likely to demonstrate refined neural control strategies, adaptation, and possibly even enhanced plastic potential. While comparative case series inherently limit generalizability, they offer unique access to the upper bounds of adaptive neurophysiology, serving as models of what the human central nervous system can achieve under extreme conditions.

In this study, we applied muscle synergy analysis, which quantifies muscle coordination patterns during movement based on multiple surface electromyography (sEMG) signals (5, 6). This method is used in a wide range of fields, such as clinical assessment of spinal cord injuries and strokes (7, 8). Recently it has been extended to sports movements such as overarm throwing (9, 10). Furthermore, synergy structure can be reorganized as a result of development or training (2), providing a powerful framework for examining the influence of use-dependent and impairment-related within the Paralympic context.

Therefore, the purpose of this study is to clarify the characteristics of muscle coordination patterns in a Paralympic gold medal wheelchair tennis player. Our findings may offer support to athletes and coaches involved in adapted sports and may also provide a foundation for future comparative or longitudinal studies aimed at understanding the boundaries and mechanisms of human adaptability.

## 2 Materials and methods

### 2.1 Participants

Two male wheelchair tennis athletes participated in this study, both affiliated with the International Tennis Federation who participate in open-class competitions. The first athlete (hereafter referred to as P1, for “Player 1”) was ranked No. 1 in the junior wheelchair tennis world rankings in 2021. In 2023, he won at both French Open and Wimbledon, becoming the youngest male champion in the history of these Grand Slam tournaments, thereby confirming his world No. 1 ranking. Subsequently, he achieved a gold medal at the Paris Paralympic Games. P1 was diagnosed with osteosarcoma at the age of nine, resulting in surgical removal of a portion of his left hip and femur. Additionally, part of his left abdominal muscle was removed due to pulmonary metastasis. Due to the impairment, weak voluntary muscle contractions were observed in the surroundings of the resected area.

The second athlete, referred to as P2, was globally ranked in the 90s and had a diagnosis of congenital spina bifida. Owing to the impairment, paralysis was present in the part of the lower limb and lower abdomen, with spasticity also observed. The trunk and lower limbs were not completely paralyzed, and motor function remained at a low level, with only limited residual capability allowing independent ambulation without assistive devices. Substantial lower-limb weakness was evident, and somatosensory function was diminished in regions below the lower leg, particularly. During gait, P2 exhibited a gait pattern characteristic of individuals with spina bifida, in which the support leg was maintained in a flexed knee position and compensated for reduced limb propulsion by generating the swing phase primarily through trunk rotation. Thus, they could walk independently and had intact upper limb function, but had different types of disabilities. Each had accumulated over 5 years of competitive athletic experience and ranked within the top ten in their respective national rankings. The participants provided written informed consent to participate in this study. All experimental procedures were conducted in accordance with the Declaration of Helsinki and were approved by the Local Ethics Review Committee of the Graduate School of Arts and Sciences, The University of Tokyo (Approval number: 900-4).

### 2.2 Procedure

Participants first completed static and dynamic stretching, followed by a sport-specific warm-up that included wheelchair propulsion, directional change, stroke practice, and serving movements. Assuming first-serve scenario, P1 completed 5 trials and P2 completed 10 trials in total. For P1, only three trials could be used because (1) synchronization between video and EMG signals was successful, and (2) the attempts were valid first serves (i.e., neither faults nor toss errors). Although EMG signals were available for all five attempts, the trials without synchronized video were excluded as ball speed and phase identification could not be verified. For P2, six out of ten trials met the same criteria, and EMG signal quality was additionally confirmed through visual inspection for noise contamination. To maintain consistency between players and to minimize potential fatigue effects, the earliest three valid trials from P2 were selected for analysis. During each serving trial, surface electromyography (sEMG) was used to measure whole-body muscle activity in order to quantify whole-body muscle coordination through muscle synergy analysis. After all serve trials were completed, maximum voluntary isometric contraction (MVC) of 2 sets of 3 s was performed to compare the muscle activity of both athletes. Simultaneously, high-speed video recordings were captured from lateral and posterior perspectives. The two video streams were synchronized with EMG signals using a light-emitting diode (LED) signal and the analog output to the data acquisition system, calibrated to a reference voltage of 5 V. This synchronization enabled precise extraction of EMG data corresponding to the phases of the serve movement.

### 2.3 Instrumentation and data acquisition

#### 2.3.1 Digitizing the ball position

Ball trajectories were recorded using high-speed cameras (DSC-RX10M4, SONY, Japan; 960 fps) positioned laterally to each player. Each recording setup was arranged to ensure unobstructed capture of the ball's trajectory from the toss through post-impact. A 0.2 m calibration frame was utilized to convert pixel data into real-world coordinates system. No markers were attached to the ball or players. The ball was tracked, and its position was identified using DeepLabCut (11), a markerless pose estimation system based on deep neural networks (12). Before training, the movies were trimmed from the ball toss to the end of the follow-through. From each player, 40 video frames (covering toss to follow-through) were manually selected using Adobe Premiere Pro to ensure visibility of the ball, particularly near the moment of impact. After 50,000 training iterations, we used these models to track and digitize the ball position across all the remaining trials. Accordingly, subsequent analyses were conducted using the data obtained from both athletes, encompassing all six of their serve trials. A fourth-order low-pass Butterworth filter with a cut-off frequency of 10 Hz was applied to the position data. Ball velocity was then computed by differentiating the position coordinates between two separate frames. Furthermore, to evaluate the consistency of the serves, the coefficient of variation (CV) for serve speed was calculated.

#### 2.3.2 Electromyography (EMG)

We recorded sEMG signals using Trigno Wireless System (DELSYS; Boston, MA, USA). Electrodes were placed on 14 muscles: dominant side of upper limb, triceps brachii (TB), biceps brachii (BB), flexor carpi radialis (FCR), extensor carpi radialis (ECR) and both side of the body external oblique (EO), lower trapezius (lTz), erector spinae (ES), tibialis anterior (TA), rectus femoris (RF). Electrode placement followed the recommendation from Surface Electromyography for the Non-Invasive Assessment of Muscles (SENIAM) (13) to minimize crosstalk from the adjacent muscles. All EMG data were analog output at 1,000 Hz and recorded on a PC using an analog-to-digital converter (Powerlab 16/35, AD Instruments, Castle Hill, NSW, Australia). First, each muscle activity was normalized by MVC. As the two athletes had different types of impairment, it was possible that MVC was not performed correctly; therefore, comparisons were primarily made within the subject. Next, to extract muscle synergy, EMG normalized to peak values for each muscle across all trials was used.

### 2.4 Muscle synergy analysis

#### 2.4.1 EMG pre-processing

We analyzed raw EMG data based on the toss to follow-through of each serve. EMG data analysis focused on the movement of the tennis serve divided into the following four key events: the ball toss (the moment when the ball leaves the hand), the takeback phase (from the ball toss to the ball impact), the impact (the moment of ball impact), and the follow through phase (from impact to the maximal shoulder adduction). The data were high-pass filtered (40 Hz, zero-lag fourth-order Butterworth) (9, 14), full-wave rectified, and low-pass filtered (10 Hz, zero-lag fourth-order Butterworth (15, 16). Subsequently, the rectified data was normalized to the peak values for each muscle across all trials. Data were time-interpolated to 201 points for each trial (17–19) yielding the EMG matrix of 14 muscles × 201 data points for synergy analysis.

#### 2.4.2 Muscle synergy analysis

Muscle synergies were extracted using a non-negative matrix factorization (NMF) algorithm (6, 20). The muscle activation pattern (*M*) is composed of a linear combination of muscle synergies $\left(W_{i}\right)$ and synergy activation coefficient $\left(C_{i}\right)$ as expressed in Equation 1:

$$\begin{array}{c} M=\overset{N}{\underset{i=1}{\sum }}W_{i}C_{i}+\;\varepsilon \;(W_{i}\geq 0,C_{i}\geq 0) \end{array}$$ (1)

where $W_{i}$ (m×n matrix, where *n* is the number of synergies) is the time-invariant weighting of each muscle components of synergy *i*, $C_{i}$ (n×t matrix) is the synergy activation coefficient, representing temporal activation patterns of synergies, and ε is the residual error matrix.

To determine the appropriate number of muscle synergies, we computed the variability accounted for (VAF) using Equation 2, which compares the original EMG data $\left(EMG_{o}\right)$ to the reconstructed data $\left(EMG_{r}\right)$. The number of muscle synergies for each dataset was defined as the minimum number of synergies required to exceed 90% of the VAF (19).

$$\begin{array}{c} \mathrm{VAF}=1-\frac{{\left(EMG_{o}-EMG_{r}\right)}^{2}}{EMG_{o}^{2}}\times 100 \end{array}$$ (2)

After extraction, each synergy was sorted according to cosine similarity (21). This functional sorting was performed by comparing each synergy's ***W*** and/or ***C*** vectors to those of a reference subject using cosine similarity. An iterative clustering process grouped synergies with the highest similarity values. If a synergy from one participant was initially assigned to multiple groups, the one with the highest cosine similarity was retained to ensure consistent classification.

To verify that the synergies extracted from P1 and P2 were not attributable to noise or random variability, we performed a cross-validation procedure. This approach follows previous studies on the robustness of muscle synergy extraction (22, 23). The minimum number of synergies satisfying VAF > 90% required four synergies for P1, whereas three synergies were required for P2. Specifically, the synergy vectors (W) identified in P2 were fixed, and the activation coefficients (*H*) were estimated using the original EMG data of P1. The reconstructed EMG signals of P1, obtained from the fixed *W* and optimized *H*, were then compared with the original EMG signals to assess the reconstruction accuracy using VAF. This procedure was repeated 20 times with different random initializations of *H*. The average VAF across the 20 iterations exceeded 90% for both P1 and P2, indicating that the extracted synergies were minimally affected by noise.

#### 2.4.3 Muscle synergy sparseness

Briefly, there are two patterns in the composition of synergy vectors: one in which only a few muscles contribute, and another in which all muscles contribute equally. The former can be viewed as having high sparseness, while the latter reflects low sparseness, which may indicate the possibility of merging or fractionation of synergies (2). Therefore, we calculated sparseness (φ) using the following (Equation 3),

$$\begin{array}{c} \varphi =\frac{\sqrt{n}-\frac{\sum_{i=1}^{n}|w_{i}|}{\sum_{i=1}^{n}w_{i}^{2}}}{\sqrt{n}-1} \end{array}$$ (3)

where $W_{i}$ is the ith muscle component of the *W* synergy vector, and *n* = 14 is the number of muscles in the vector. A highly sparse vector with a single non-zero component has φ=1; a non-sparse vector with equal components across all muscles has φ=0. In other words, High sparseness indicates few muscles dominate the synergy (localized control), while low sparseness represents distributed control.

#### 2.4.4 Merging and fractionation of muscle synergies

To understand the P1 and P2 synergy vector in more detail, we calculated the merging and fractionation index of muscle synergies (7). Since it has been shown that merging and fractionation can occur during the development of running movements in sports movements as well (2), we calculated and evaluated these indices because they may reflect differences in impairment or competition level.

Therefore, to investigate whether the synergy of P1 can be explained by the linear combination of multiple synergies of P2, we modeled the merging synergy as shown in (Equation 4) (2, 7),

$$\begin{array}{c} {\vec{W}}_{i}^{p1}\approx \overset{N^{p2}}{\underset{i=1}{\sum }}m_{k}^{i}{\vec{W}}_{k}^{p2}\;(m_{k}^{i}\geq 0,\;i=1\ldots N^{\;p2}) \end{array}$$ (4)

Where ${\vec{W}}_{i}^{\;p1}$ is the ith merged muscle synergy, $m_{k}^{i}$ is a non-negative coefficient that scales the kth synergy in the merging, and ${\vec{W}}_{k}^{\;p2}$ is the kth synergy to be merged, $N^{\;p2}$ is the number of synergies contributing to the merging. The coefficient of $m_{k}^{i}$ was calculated through a non-negative least squares fit (using Matlab function “lsqnonneg”) after ${\vec{W}}_{i}^{\;p1}$ and ${\vec{W}}_{k}^{\;p2}$ were normalized to unit vectors. Synergy merging was identified when $N^{\;p2}\geq 2$, $m_{k}^{i}\geq 0.2$ for all *k*, and the cosine similarity between $\overset{N^{p2}}{\underset{i=1}{\sum }}m_{k}^{i}{\vec{W}}_{k}^{p2}$ and ${\vec{W}}_{i}^{p1}$ was ≥0.8.

Among these synergies, to evaluate whether the P1 synergies could be approximated by the combination of multiple synergies of P2, we calculated the cosine similarity between the synergies of P1 and the multiple combinations of P2.

Contrary to merging, we calculated P2 synergies fractionation to P1 synergies according to (Equation 5).

$$\begin{array}{c} {\vec{W}}_{i}^{p2}\approx \overset{N^{p1}}{\underset{i=1}{\sum }}m_{k}^{i}{\vec{W}}_{k}^{p1}\;(m_{k}^{i}\geq 0,\;i=1\ldots N^{\;p1}) \end{array}$$ (5)

Where ${\vec{W}}_{i}^{\;p2}$ is the ith merged muscle synergy, $m_{k}^{i}$ is a non-negative coefficient that scales the kth synergy in the merging, and ${\vec{W}}_{k}^{\;p1}$ is the kth synergy to be merged, $N^{\;p1}$ is the number of synergies contributing to the merging. The fractionation coefficient was calculated using the same procedure as merging, and the muscle synergy fractionation was modeled using the same criteria.

## 3 Results

### 3.1 Serve velocity and kinematics

**Figure 1:**
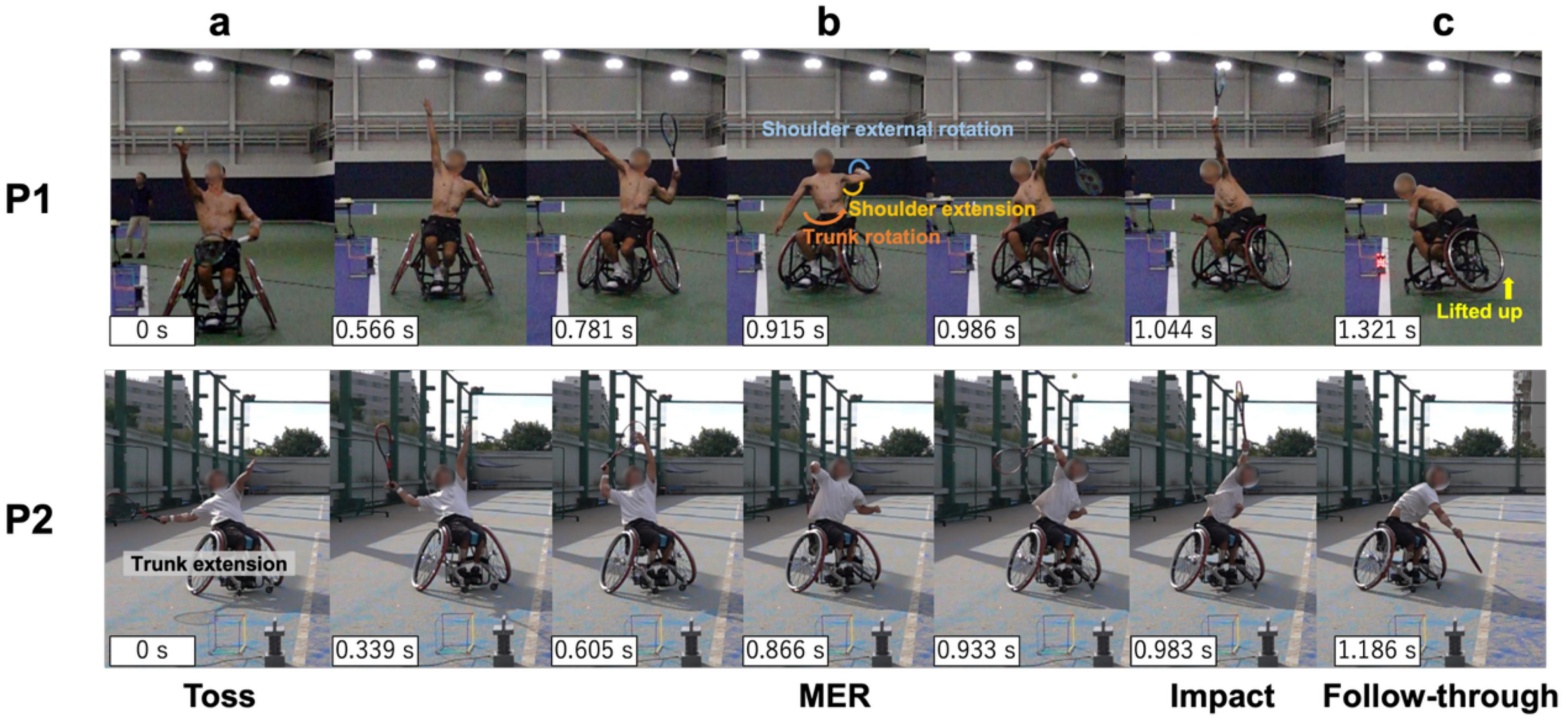
Sequence of serve movement. The camera was placed in front of the players, at face height, before the start of the experiment. They were also placed so that the LED lamp and calibration frame could be seen in each camera frame. The figure consists of seven consecutive photographs, from the ball toss to the follow-through. The ball toss is designated as 0 s, and the actual time for each frame is indicated in the lower left corner of the photograph.

Serve velocities were 167 ± 11.3 km/h (CV = 6.8%) for P1 and 80.0 ± 8.9 km/h (CV = 11.1%) for P2. High-speed footage showed P1 wheelchair facing directly position across the court; from impact to follow-through, one wheel lifted off the ground, and the wheelchair rotated. In contrast, the P2 positioned the wheelchair diagonally and exhibited more trunk extension than rotation during the takeback (Figure 1a).

### 3.2 EMG

**Figure 2:**
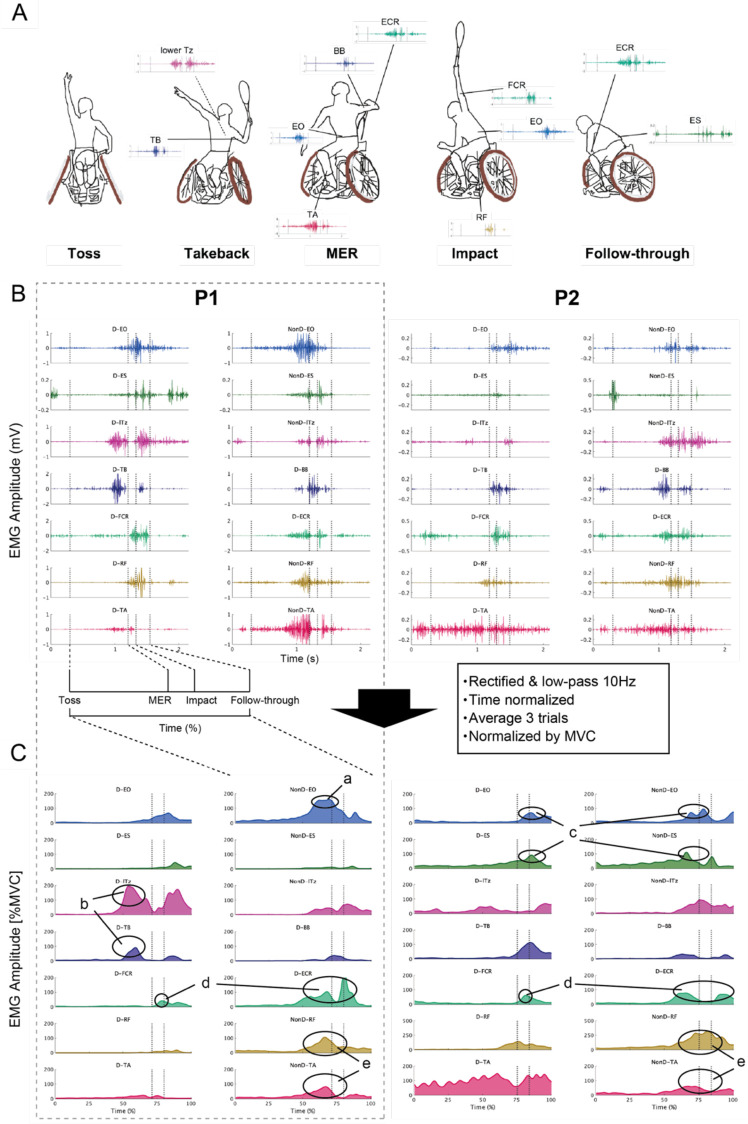
EMG data of tennis serve movement. **(A)** The main muscle activities that were activated in response to the serving movement are shown. **(B)** Examples of representative muscle activity during the serving movements of P1 and P2; EMG data were high-pass filtered at 40 Hz. The D or NonD means dominant or non-dominant side of arm. The vertical axis is shown as EMG amplitude (mV), and the horizontal axis is shown in time (s). The EMG data were plotted from 0.3 s before the start of the toss to 0.6 s after the end of the follow-through. Muscle synergy analysis was performed from the start of the toss to the end of the follow-through. **(C)** The EMG data shown in **B** were time normalized after rectified and low-pass 10 Hz processing and normalized by maximum voluntary isometric contraction (MVC) for each participant after an ensemble average of three trials. The vertical axis is shown as a percentage of the MVC, and the horizontal axis is time normalized from the start of the toss to the end of the follow-through. The high value of %MVC for the lower limbs in P2 may be due to weak voluntary contraction by impairment. During the serve, the feet were fixed to the wheelchair, so it is thought that strong muscle activity occurred passively. Note that while Figure **C** shows EMG waveforms normalized to MVC for comparing muscle activity levels, muscle synergy analysis uses EMG normalized to the maximum value of each muscle.

Figure 2A shows the major muscles activity during each phase of the tennis serve, and Figure 2C shows the EMG envelope normalized by MVC for each player. The characteristics of both players are described below. P1 exhibited pronounced activation of the lTz and TB muscles prior to maximum external rotation (MER), while P2 demonstrated little activity during this phase (Figure 2C-b). The activation timings of the trunk muscles, EO and ES were comparable between the two participants, with the non-dominant side being active during the pre-impact phase, followed by an increase in activity of the dominant-side trunk muscles. However, the activity levels of the trunk muscles showed a difference between the two athletes, with higher activity levels of both sides of EO than ES in P1, whereas ES activity levels were equivalently high with EO in P2 (Figure 2C-c). For the forearm muscles, both players displayed generally similar activation timings, with peak muscle activity for FCR was observed at the impact, and greatest activity for ECR occurred just before MER and post-impact (Figure 2C-d). The ECR had a once-lower activity level during the phase between MER and impact. In both athletes, the activities of the lower extremity muscles (RF and TA, Figure 2C-e) on the non-dominant side were greater than the dominant side except for TA in P2. Because P1 dominant (left) hip joint was affected by his disability; TA had a smaller muscle activity compared to the non-dominant side.

### 3.3 Muscle synergies

**Figure 3:**
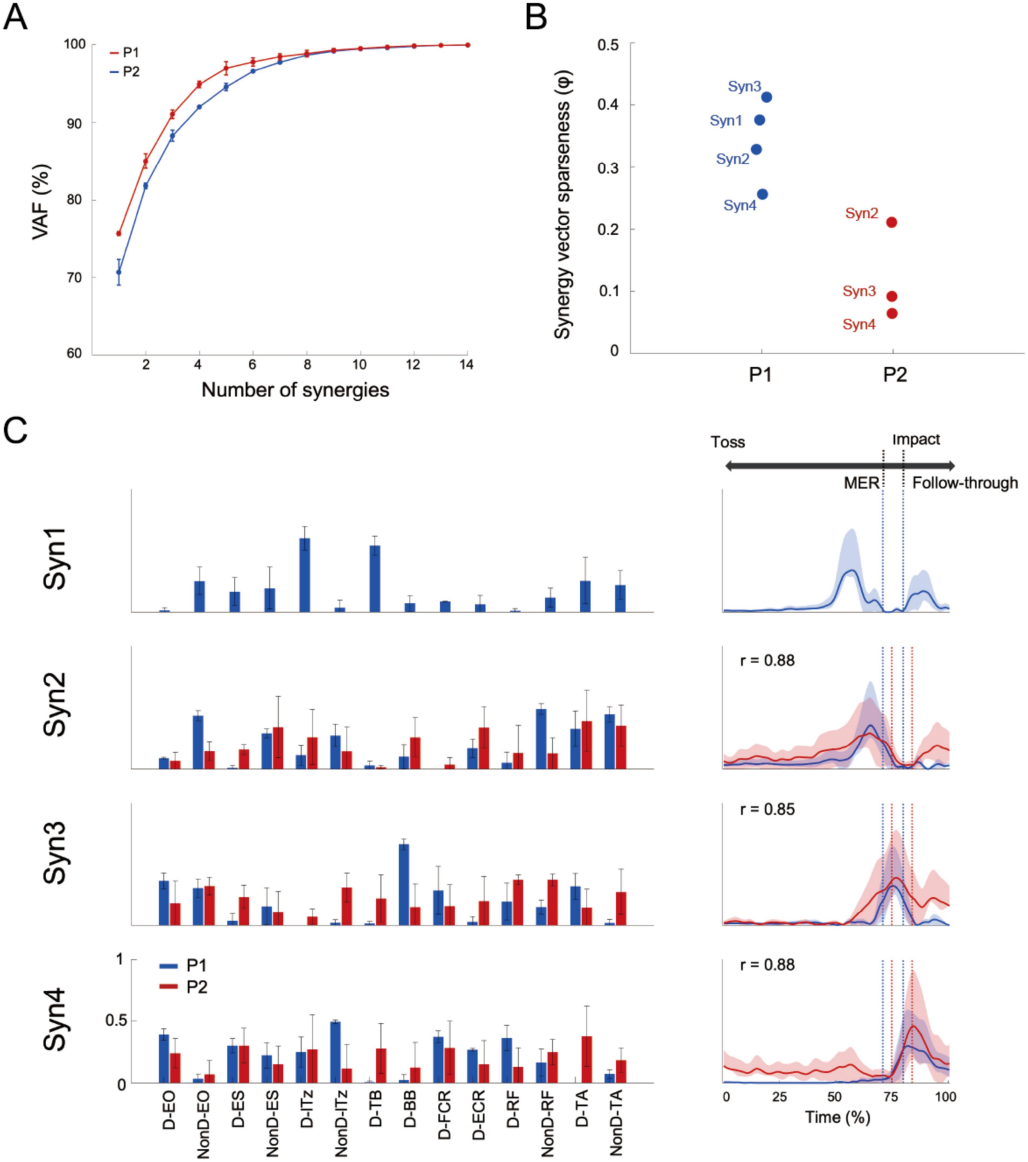
Muscle synergies and activation coefficient of serve movement. **(A)** The mean VAF values for P1 and P2 are shown, and the bars represent the standard deviation. The number of muscle synergies with VAF > 90% was four for P1 and three for P2. **(B)** The values of sparseness of each extracted synergy vector for P1 and P2 are shown. The mean value of all synergies was 0.343 for P1 and 0.123 for P2, indicating that sparseness was lower for P2 than for P1. **(C)** Muscle synergies extracted from the 14 muscles. Blue and red indicate P1 and P2, respectively. The correlation coefficients (*r*) show the values for pairs with the highest similarity between P1 and P2. Syn1 was specific for P1 and was mainly contributed by lTz and TB, with the greatest activity in the takeback phase.

For each player, the minimum number of muscle synergies required to satisfy the VAF criterion (VAF > 90%) was four for P1 (VAF = 92.0 ± 0.1%) and three for P2 (VAF = 91.1 ± 0.6%) (Figure 3A). Pairs with the highest cosine similarity were matched across participants revealing that synergy 1 in P1 was specific to that individual (*r* < 0.73 for any pairwise comparison). All other synergy combinations exhibited correlation coefficient exceeding *r* > 0.85. Synergy 1 in P1 was primarily characterized by strong contributions from the lTz and TB, with peak activation occurring during the takeback phase preceding MER. Synergy 2 was predominantly composed of the lTz and ES, showing maximal activity immediately prior to MER. Although the specific muscles comprising synergy 3 varied between participants, the temporal profile of this synergy consistently demonstrated peak activity between MER and ball impact, indicating its involvement in the acceleration phase of the serve. Synergy 4 reached its maximum activation at the moment of impact and was primarily influenced by bilateral ES, the dominant-side EO, and upper extremity muscles.

### 3.4 Merging and fractionation of muscle synergies

**Figure 4:**
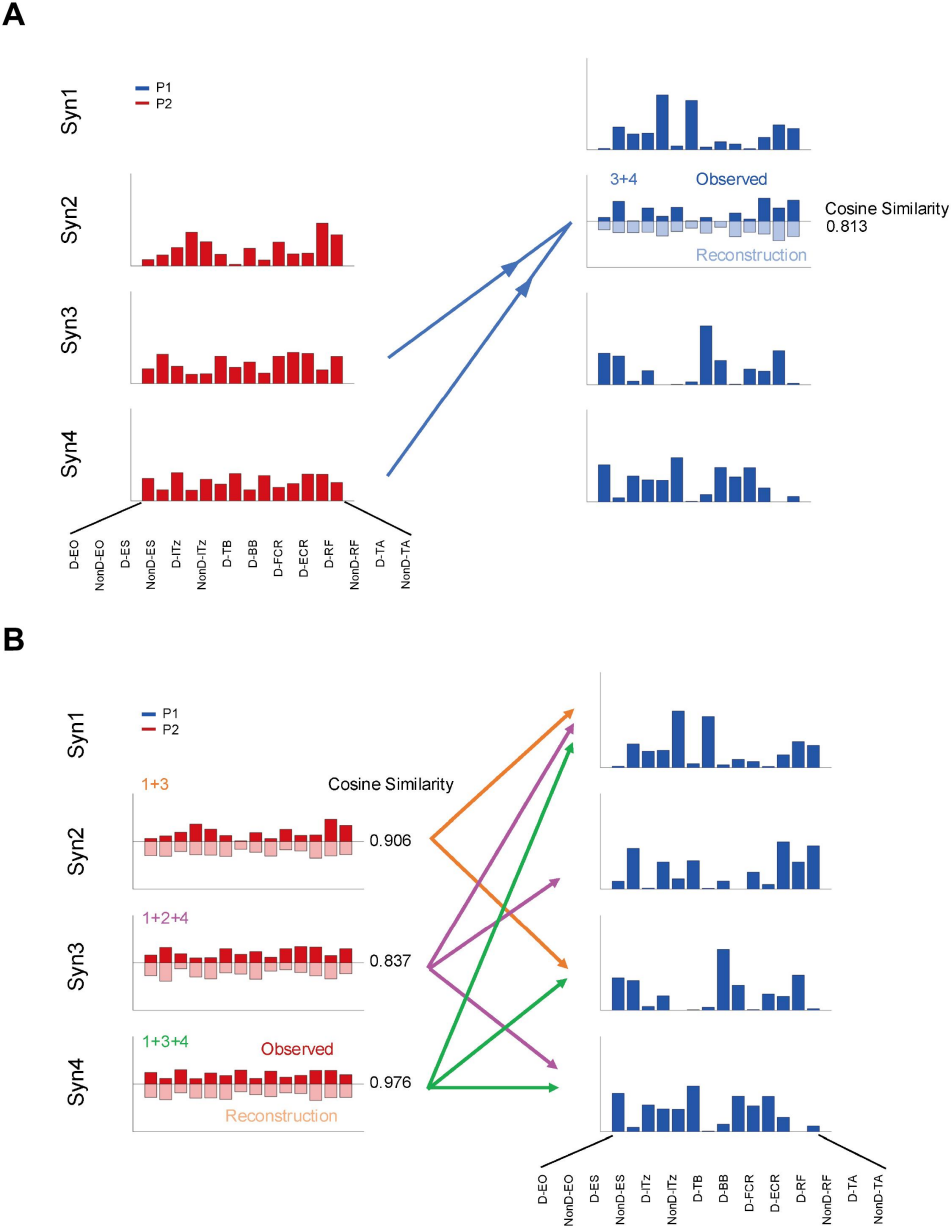
Merging and fractionation of muscle synergies. **(A)** Merging of muscle synergy of P2, showing that it could be reconstructed a synergy vector similar to Syn2 in P1 by multiple synergies in P2. The cosine similarity between original and reconstructed synergy vector of Syn3 + 4 in P2 was 0.81. Furthermore, the evidence that Syn1 in P1 cannot be approximated by any combinations of the synergy vectors in P2 strengthens the possibility that Syn1 is specific to P1. **(B)** The synergy vector of P2 could be explained by multiple linear combinations of P1 (cosine similarity between original and reconstructed vectors = 0.84–0.98). Thus, the synergy of P2 was fractionated into multiple synergies in P1 (e.g., Syn2 in P2 was split to Syn1 and 3 in P1).

We found that Syn3 + 4 in P2 could be approximated by the muscle synergy vector of Syn2 in P1 (*r* = 0.813). However, Syn1 in P1 could not be approximated by any of the synergy combinations in P2, which means that Syn1 in P1 was specific (Figure 4A).

The sparseness of the muscle synergy vector averaged 0.343 and 0.123 for P1 and P2, respectively, with the weightings of P2 was more uniformly distributed than P1. On the other hand, for fractionation of muscle synergy, Syn2 in P2 could be reconstructed as combination of Syn1 + 3 from P1, Syn3 in P2 to Syn1 + 2 + 4 from P1, and Syn4 in P2 to Syn1 + 3 + 4 from P1 in a fractionation of muscle synergy (Figure 4B).

## 4 Discussion

This study aimed to elucidate the characteristics of muscle coordination patterns during the serve motion of the Paralympic gold medalist wheelchair tennis player. The serve velocity of P1 was approximately twice that of P2 and P1's EO muscle exhibited greater activation than P2's from takeback to ball impact phase (Figure 2C-a). The muscle synergy analysis revealed four distinct synergies in P1 and three in P2, and the synergy associated with the takeback was specific for P1 (Figure 3C). These results are thought to reflect not only differences based on competitive level but also the influence of impairment. In particular, the appropriate recruitment of residual trunk and lower limb muscles may vary according to impairment characteristics.

### 4.1 The variations of trunk movement depend on impairment

Based on video analysis, P1 exhibited noticeable upper trunk rotation, shoulder extension, and MER during the takeback phase (Figure 1b). This movement may generated sufficient rotational energy to lift one wheel of the wheelchair during the follow-through phase (Figure 1c). These results suggest that the generation and transfer of rotational energy through trunk rotation and flexion may be a key factor in increasing the serve speed in wheelchair tennis. In tennis serve, the efficient transfer of kinetic energy to the ball is critical for achieving high ball velocity (24). This highlights the importance of coordinated movement across multiple body segments—the kinematic chain (25, 26). In wheelchair players, the absence of leg drive necessitates the generation of high velocity serves from an inherently unstable seated position. Under these conditions, trunk function—particularly rotational movement—may act as the initial driver in the formation of the kinematic chain. Regarding the shoulder joint, it has been demonstrated that improving internal and external rotation range of motion while reducing muscle imbalances enhances ballistic torque generation (27). This indicates that achieving both shoulder joint stability and a wide range of motion improves overhead performance and suggests that shoulder joint function, which connects the trunk and upper limbs, is involved in energy transfer. In contrast, P2 exhibited trunk extension during the ball toss phase (Figure 1a), followed by trunk flexion toward impact. This strategy suggests that individuals with trunk paralysis may compensate for limited trunk rotation by shifting their center of gravity and performing extension movements using preserved functions such as the upper limbs and head.

### 4.2 Coordination strategies depend on impairment characteristics

In P1, the non-dominant EO exhibited greater activation than the ES activity. In able-bodied tennis, when comparing advanced and intermediate players during the acceleration phase of a flat serve, it was reported that EO activity was greater in advanced players, while ES was similar. In this study intermediate players exhibited greater trunk extension than advanced (28). This study further suggested that EO and ES activation levels affect the efficiency of the tennis serve, as EO and ES have an agonist—antagonist relationship. As observed in P1, wheelchair tennis athletes with almost preserved trunk function may rely on the balance between EO and ES activity as a key indicator, similar to patterns reported in able-bodied tennis. In the case of P2, similar activation levels of the EO and ES were observed, indicating a pattern of greater co-contraction compared with P1 (Figure 2C-c). Apparently, elevated ES activation during the acceleration phase of the serve may seem to interfere with the coordinated trunk motion required for effective energy transfer. However, the relatively high ES activity observed in P2 likely reflects a compensatory postural control strategy aimed at increasing trunk stiffness in the context of paralysis affecting the lower limbs and lower trunk. Milosevic et al. (8) demonstrated that people with higher-level thoracic spinal cord injury exhibit greater co-activation within trunk muscle synergies during seated reaching. This likely helps them stabilize the upper body by simultaneously recruiting several residual muscles. These findings are consistent with the interpretation that P2 employed increased ES co-activation to maintain seated balance while executing the serving motion.

In addition, the level of muscle activity should be interpreted with caution due to the limitation of MVC methodology. Previous studies have shown that muscle activity exceeding MVC during high-velocity, sport-specific movements, likely due to mechanisms such as the stretch reflex or the capacity to generate greater force under dynamic conditions compared to static (isometric) MVC testing (29). Especially in people with pain or impairments, as in the present study, accurate MVC execution may be compromised (30, 31). Therefore, it is important to note that this study focused on differences in muscle activity levels within subjects rather than between subjects.

In both athletes, lower limb muscle activity was higher in the non-dominant RF and TA (Figure 2C-e). These results suggest that the fixed positioning of the lower limbs within the wheelchair may facilitate explosive trunk flexion and rotation through residual motor function. Previous study has shown that fixing the torso and lower limbs with straps improves explosive movement performance, such as sprinting (32). Particularly, the greater activation of the non-dominant RF was observed in P2 than P1. This may indicate that RF acted as a driving force for trunk flexion from a position of hyperextension.

### 4.3 Functional role of extracted muscle synergies during serve motion

Muscle synergy analysis identified four distinct muscle synergies (Syn1–4) in P1 and three synergies (Syn2–4) in P2 (Figure 3). Syn2–4 were determined to be similar muscle synergies between both athletes based on cosine similarity. Regarding Syn2 in P1, the synergy vectors were characterized by contributions from the non-dominant EO and RF. However, P2's Syn2 exhibited a different pattern from that of P1, with ES contributing. This pattern may reflect the level of residual function in the trunk and lower limbs, suggesting that coordination strategies differ according to the degree of paralysis and the type of impairment. When the trunk and lower limbs can be used normally or almost normally, the non-dominant EO and RF may play an important role in maximizing MER, as observed in P1. Furthermore, during the transition from MER to impact regarding Syn3, BB had a greater contribution in P1 than P2, while TB had a greater in P2 than P1. These results may be related to the efficiency of energy transfer and stabilization of the elbow joint. In tennis serves, the TB has shown to contribute more to elbow joint stability through co-contraction than to generate elbow extension torque (33). Additionally, in overhead throwing motions, elbow extension torque was reported to decrease during the acceleration phase compared to the cocking phase (34). Syn1 was specific to P1 and was associated with trunk rotation during the takeback phase. It may reflect a strategic neuromuscular adaptation for optimizing residual trunk and upper limb function while seated in a wheelchair. This difference between P1 and P2 is further supported by the individual EMG patterns observed between P1 and P2 (lTz and TB, Figure 2C-b). The primary muscles constituting Syn1 are the upper limbs (TB and lTz), which are not directly affected by paralysis, although their neuromuscular control strategies may be influenced by their own impairments.

### 4.4 Sparseness of muscle synergies

More synergies were extracted from P1 than P2 and, each synergy in P1 was sparse compared to those of P2 (Figure 3B). The movement patterns of both athletes may be adapted to their respective movement of the tennis serve based on their impairment characteristics. Specifically, P1 retains much physical function, allowing him to selectively activate the muscles to perform the tasks required at each phase of the serve. In the case of P2, who has paralysis affecting a large area of the lower limbs and trunk, may achieve each phase of the serve by extensively recruiting muscles beyond the agonist muscles—such as for maintaining posture during the serve—and by utilizing as many available muscle groups as possible. In forehand strokes, it has been shown that the level of co-contraction in the upper limbs clearly shifts from the backswing to the follow-through. This coordinated interaction has been reported to be essential for smooth movement, ensuring two elements: force exertion and joint stability (35). The sparseness of each synergy in P1 may enable more precise and phase-specific control in serve. In contrast, P2 showed simultaneous activation of multiple muscles due to the impairment, suggesting that a wider muscle control strategy adjusted to the level of paralysis is needed.

To further investigate, we examined whether Syn1 from P1 could be approximated by linear combinations of multiple synergies from P2. However, no combination of synergies from P2 could approximate Syn1 from P1 (Figure 4A). This supports the result that Syn1, associated with the takeback phase, was specific to P1.

Additionally, as shown in Figure 4B, Syn2–4 from P2 could be explained as a fractionation of the synergies of P1. This is reasonable given the small number of synergies extracted from P2 and that they were less sparse than those of P1. This suggests that P2 controlled many muscles during each phase of movement. Considering the effect of P2's impairment, co-contraction of the trunk muscle groups may contribute to performance, such as maintaining balance and stability. While prior research involving patients with severe stroke was less sparse on the stroke-affected arm and some synergies were merging from the unaffected-side (7). Thus, simultaneous activation of many muscles may affect the achievement of smooth movements. Similarly, in whole-body rhythmic movements among healthy individuals, dancers who are skilled at manipulating their bodies have lower levels of co-contraction (36). We demonstrated muscle coordination patterns in the serve motion in wheelchair tennis by using muscle synergy analysis, and that excessive co-contraction during the serve may negatively affect serve performance.

### 4.5 Limitations

This study has several limitations that should be noted. First, as this study is a case study focusing on a Paralympic gold medalist, these findings may not be applicable to the broader population of wheelchair tennis players. Furthermore, the two athletes in this study had different disabilities, which makes it difficult to attribute differences in neuromuscular control strategies solely to performance level. Although wheelchair tennis is among the most prominent parasports globally, there is a lack of research on movement strategies and the interaction between impairments and residual motor function (37). Although techniques and motor control strategies depend on individual impairments or playing styles, conducting a wide-ranging survey of the neuromuscular functions of wheelchair tennis players will provide a basis for considering appropriate classification or training strategies. To the best of our knowledge, this is the first study to characterize the muscle coordination patterns during the serve motion in a Paralympic gold medalist in wheelchair tennis. Second, without full kinematic data, any relationships between muscle synergies and joint torques (e.g., energy transfer) remain interpretive. In the future, conducting comprehensive biomechanical and electromyographic analysis across individuals with diverse range of impairments may facilitate the development of optimal training methods and wheelchair settings that account for impairments.

### 4.6 Practical application

In the Open class of wheelchair tennis, players have many different types and levels of impairment, which leads to large differences in trunk and lower-limb function. Therefore, coaches need to understand each player's functional abilities and adjust their coaching accordingly. In particular, it is important to focus on trunk function and first ensure a stable sitting posture. This often requires wheelchair adjustments, so working with equipment specialists is helpful. After postural stability is achieved, training to improve trunk rotation and shoulder stability becomes especially important.

## 5 Conclusion

Even among wheelchair tennis players classified within the same competition class, the level of residual function, particularly trunk function, plays a critical role in determining the muscle coordination patterns required for serve. The Paralympic gold medalist, who possessed greater residual trunk function and competed at a higher performance level showed greater activity in the lTz and TB during the takeback, and in the EO from the MER to the impact phase. Furthermore, muscle synergy analysis revealed that the Paralympic gold medalist exhibited a specific synergy involved in the takeback phase, suggesting that it contributed to optimizing the takeback motion. In contrast, the athlete with lower residual trunk function appeared to enhance stability by recruiting a greater number of muscles. These findings emphasize that in wheelchair tennis, serve performance would rely not only on muscular strength but also on muscle coordination, especially between the trunk and upper limbs. Clinicians and coaches should consider the residual functions associated with disability when facilitating appropriate movement patterns, and they may also need to optimize wheelchair settings when necessary.

## Data Availability

The raw data supporting the conclusions of this article will be made available by the authors, without undue reservation.
