# Correction: Synergistic muscle coordination of the Paralympic wheelchair tennis champion

**DOI:** 10.3389/fspor.2026.1878833

**Published:** 2026-06-11

**Authors:** Nadaka Hakariya, Takuya Murakami, Keiichi Ishikawa, Daiki Yamasaki, Naotsugu Kaneko, Kimitaka Nakazawa

**Affiliations:** 1 Department of Life Sciences, Graduate School of Arts and Sciences, The University of Tokyo, Tokyo, Japan; 2 Department of Rehabilitation for the Movement Functions, Research Institute of National Rehabilitation Center for Persons with Disabilities, Tokorozawa, Japan; 3 Japan Society for the Promotions Science, Tokyo, Japan

**Keywords:** wheelchair tennis, Paralympic athletes, muscle synergy, impairment-related adaptation, neural plasticity

Authors Keiichi Ishikawa and Daiki Yamasaki were omitted as authors in the published article. The correct author list reads:

Nadaka Hakariya, Takuya Murakami, Keiichi Ishikawa, Daiki Yamasaki, Naotsugu Kaneko, Kimitaka Nakazawa

The **Author Contributions** statement has been corrected to read:

NH: Conceptualization, Data curation, Formal Analysis, Funding acquisition, Investigation, Methodology, Writing – original draft, Writing – review & editing. TM: Investigation, Writing – review & editing. KI: Investigation, Writing – review & editing. DY: Investigation, Writing – review & editing. NK: Conceptualization, Funding acquisition, Methodology, Visualization, Writing – review & editing. KN: Conceptualization, Methodology, Project administration, Supervision, Writing – review & editing.

The omitted authors were originally thanked in the **Acknowledgments** instead of appearing the author list. The correct **Acknowledgments** is:

“The authors thank the Japan Broadcasting Cooperation (NHK) for giving us the opportunity of experiment.”

A correction has been made to the section **2 Materials and Methods**, *2.4 Muscle synergy analysis*, “2.4.1 EMG pre-processing”:

“We analyzed raw EMG data based on the toss to follow-through of each serve. EMG data analysis focused on the movement of the tennis serve divided into the following four key events: the ball toss (the moment when the ball leaves the hand), the takeback phase (from the ball toss to the ball impact), the impact (the moment of ball impact), and the follow through phase (from impact to the maximal shoulder adduction). The data were high-pass filtered (40 Hz, zero-lag fourth-order Butterworth) (9, 14), full-wave rectified, and low-pass filtered (10 Hz, zero-lag fourth-order Butterworth (15, 16). Subsequently, the rectified data was normalized to the peak values for each muscle across all trials. Data were time-interpolated to 201 points for each trial (17–19) yielding the EMG matrix of 14 muscles × 201 data points for synergy analysis.”

Equation 2 in **2 Material and methods**, *2.4 Muscle synergy analysis, “*2.4.2. Muscle synergy analysis”, Paragraph 2 was erroneously given as:

(2) $$\;\;\;\begin{array}{c} VAF=\frac{{\left(EMG_{o}-EMG_{r}\right)}^{2}}{EMG_{o}^{2}}\times 100\;\;\;\;\;\;\;\;\; \end{array}$$

The correct equation is:

(2) $$\;\;\begin{array}{c} \mathrm{VAF}=1-\frac{{\left(EMG_{o}-EMG_{r}\right)}^{2}}{EMG_{o}^{2}}\times 100 \end{array}\;\;\;\;\;\;\;\;$$

Additionally, the in-text citations for Equations 3–5 were missing the links to the equations.

A correction has been made to the section **3 Results**, 3.*4 Merging and fractionation of muscle synergies*, Paragraphs 1 and 2:

“We found that Syn3 + 4 in P2 could be approximated by the muscle synergy vector of Syn2 in P1 (*r* = 0.813). However, Syn1 in P1 could not be approximated by any of the synergy combinations in P2, which means that Syn1 in P1 was specific (Figure 4A).

The sparseness of the muscle synergy vector averaged 0.343 and 0.123 for P1 and P2, respectively, with the weightings of P2 was more uniformly distributed than P1. On the other hand, for fractionation of muscle synergy, Syn2 in P2 could be reconstructed as combination of Syn1 + 3 from P1, Syn3 in P2 to Syn1 + 2 + 4 from P1, and Syn4 in P2 to Syn1 + 3 + 4 from P1 in a fractionation of muscle synergy (Figure 4B).”

The original version of this article has been updated.

